# The effects of exergaming on sleep quality: a systematic review and meta-analysis of randomized controlled trials

**DOI:** 10.3389/fdgth.2026.1699626

**Published:** 2026-02-09

**Authors:** Ziyu Hao, Yaxin Hu, Ke Chen, Karen Yiu, Pingping Jia, Kelvin Tsoi

**Affiliations:** 1 JC School of Public Health and Primary Care, The Chinese University of Hong Kong, Hong Kong, China; 2 Stanley Ho Big Data Decision Analytics Research Centre, The Chinese University of Hong Kong, Hong Kong, China

**Keywords:** aerobic exercise, insomnia, older adults, PSQI, virtual reality

## Abstract

**Background:**

Reduced sleep quality is a growing concern that can affect both mental and physical health in individuals of all ages. Exergames, which combine physical activity with gaming elements through digital technology, are an emergent popular intervention nowadays. This review aims to review the potential effects of exergames on sleep quality.

**Methods:**

This review is registered in PROSPERO under the identifier CRD42024601725. A systematic search of the literature was performed on February 13, 2025, across MEDLINE, EMBASE, SCOPUS, Web of Science, CENTRAL, CBM, and CNKI. Randomized controlled trials comparing the effects of exergame interventions on sleep quality were included. Standardized mean differences (SMD) with 95% confidence intervals (CI) were calculated to compare various sleep quality scales. Heterogeneity was assessed using the I² statistic and Cochran's *Q* test, and random effects models were applied in the meta-analysis where appropriate. Subgroup analyses were conducted based on participant age and the type of exergaming.

**Results:**

Our search identified a total of 800 studies, and 11 articles comprising 521 participants were included. Participants ranged in age from 9 to over 80 years old, with 41% being male. Among 9 studies comparing exergaming with no intervention, participants who received exergaming intervention showed better sleep quality [SMD [95% CI] of −0.31[−0.60, −0.01], k = 9]. Subgroup analysis indicated participants who had interventions lasting 8 weeks or longer [−0.35(−0.64, −0.06), k = 5], engaging in aerobic exercise-based exergaming experienced [−0.35 (−0.59, −0.11), k = 7], aged 60 and older [−1.38 (−2.75, −0.00), k = 2] showed significant improvements in sleep. Moreover, three studies comparing exergaming with traditional exercise showed no significant differences.

**Conclusions:**

Exergame interventions positively influence sleep quality, particularly for older adults or those with health conditions. Given their accessibility and ease of use, exergames can serve as a self-driven, long-term intervention at home.

**Systematic Review Registration:**

PROSPERO CRD42024601725.

## Introduction

Sleep quality is an increasingly recognized issue affecting individuals across all age groups. Common challenges include difficulty falling asleep, feeling tired after waking up, or even insomnia. Globally, an estimated 27% of adults experience problems initiating or maintaining sleep, with insomnia being one of the most prevalent sleep disorders. Furthermore, age was found to be directly and significantly associated with lower sleep quality, suggesting that older adults are more likely to suffer from poor sleep (1).

Poor sleep quality is strongly associated with adverse health outcomes, for both mental and physical perspectives. Adolescents with insufficient sleep are more likely to report headaches (2) and mental health challenges, such as low mood and self-harm (3). In older adults, poor sleep quality increases the risk of depression and cognitive impairment (4). Additionally, insomnia is associated with chronic conditions, including anxiety, depression, respiratory and musculoskeletal disorders, hypertension, diabetes, and cardiovascular diseases (5). Individuals with poor sleep quality are at a higher risk of developing comorbidities and experiencing a decline in quality of life (6). Although pharmacological treatments are widely used to address poor sleep quality, they often come with adverse effects, including drug tolerance and addiction, prompting the need for alternative interventions.

Exergame, a non-pharmaceutical intervention, is defined as the integration of games and exercise, where users are required to move their entire bodies to engage in physical activities in virtual reality (VR) -based or video-based games (7). Games are often praised as the ninth art, with a diverse range of types emerging, each showcasing captivating narratives, visual artistry, music, and interactive elements. As the performance of computers and mobile devices improves, the popularity of games continues to grow, making them more accessible and appealing to a wider audience. Exergames may utilize analogous mechanisms as traditional exercise to improve sleep quality. The effect sizes of active video games are comparable to those of traditional physical activities, particularly in terms of heart rate, oxygen consumption, and energy expenditure (8). Exercise broadly impacts somatic physiology through multiple physiological mechanisms. The physiological strains and depletion of body energy caused by physical training increase the need for rest, thereby promoting sleep ^(^9). Additionally, exercise stimulates the production of melatonin, a hormone that facilitates the onset of sleep, and helps reduce stress, a prevalent factor contributing to poor sleep quality (10). Like regular physical activities, exergaming also regulates body temperature, which is essential for initiating sleep (11, 12). In conclusion, exergames leverage these mechanisms, offering benefits for physical cognition and psychosocial factors, enhancing overall well-being, and potentially improving sleep quality. Compared to conventional exercise regimen, exergaming offers a more engaging and enjoyable way for individuals to maintain consistent physical activity, making it a convenient option for enhancing fitness (13).

We identified a 2024 systematic review (14) focusing on video gaming and sleep in adults. The limitation of that review is that most of the included studies were non-experimental studies, and only a qualitative analysis was conducted. Additionally, the conclusions drawn from the included studies were inconsistent. Furthermore, no other systematic review and meta-analysis exploring the impact of exergaming on sleep quality were identified. To explore feasible daily exercise methods to improve people's sleep quality, to our knowledge, we present the first meta-analysis assessing the effects of exergaming on sleep quality.

As an innovative approach, exergaming not only fosters physical activity but also supports mental well-being and improves cognitive functions, particularly for individuals with mild cognitive impairment and dementia (15). Evidence indicates an association between sleep quality and exergaming interventions (16, 18). However, it remains unclear whether exergaming can significantly improve sleep quality. Additionally, when compared to traditional exercise, the effects of exergaming remain ambiguous. Therefore, the objective of this systematic review and meta-analysis was to evaluate the effectiveness of exergames in enhancing sleep quality.

## Methods

### Study design

This systematic review and meta-analysis were conducted in accordance with the Preferred Reporting Items for Systematic Reviews and Meta-Analyses (PRISMA) guidelines (19). The review protocol was registered on PROSPERO (Registration number: CRD42024601725).

### Search strategy

A comprehensive literature search was conducted across MEDLINE, EMBASE, SCOPUS, Web of Science, CENTRAL, CBM, and CNKI, covering studies from their inception to February 13, 2025. The search strategy combined MeSH terms and free-text keywords using Boolean logic operators to connect the keywords of sleep quality and exergames. The following search terms were used: (play OR exercise OR virtual reality OR gamification OR exergame) AND (sleep OR insomnia OR polysomnography OR actigraphy OR Pittsburgh Sleep Quality Index) AND (randomized controlled trials OR controlled clinical trial OR clinical trial OR placebo OR blind* OR random*). Full search details are provided in Supplementary Table 1.

### Inclusion and exclusion criteria

Studies were included if they met the following criteria: (1) Participants: To include a wide range of participant types, there were no specific disease restrictions or age restrictions; (2) Intervention group: exergaming interventions were employed for intervention group, with clearly defined intervention strategies, such as the types of exergaming, frequency and duration; (3) Control group: a non-exergaming comparison group, such as usual care, traditional exercise or no exercise interventions were included; (4) Outcomes: the primary outcome was sleep quality, assessed by valid and appropriate methods both before and after the intervention; (5) Study design: the study design was a randomized controlled trial; and (6) there was no language restriction.

The exclusion criteria were: (1) studies for which full texts were not available, such as conference abstracts, proceedings, opinion papers, editorials, and reviews; or (2) interventions involving multiple components, such as the use of medication.

### Study selection and data extraction

Two independent reviewers screened study titles and abstracts based on the inclusion and exclusion criteria. Data were extracted by two independent reviewers (ZH and KC) into a pre-designed Excel spreadsheet, capturing study characteristics including author and year of publication, country, study setting, sample size, details of the exergaming interventions, participant demographics, and the mean and standard deviations (SD) of post-intervention sleep quality scores (20). Discrepancies were resolved through discussion or consultation with a third reviewer (YH).

### Intervention

Exergaming is typically classified into two principal categories: video-based and Virtual reality (VR)-based (21). Notable examples of exergaming devices include the Xbox Kinect, Nintendo Switch, and Samsung Gear VR. VR-based exergames immerse participants in computer-generated environments and scenarios, allowing them to interact with the virtual world as if physically present. These games typically require a VR headset that tracks head movements and displays a 3D view of the environment. To ensure user safety during movement, providing a larger and more spacious environment is essential. In contrast, video-based exergames are digital games played on electronic devices, which involve visual graphics, sound, and interactive gameplay. These games require less equipment and often include multiplayer modes, facilitating social interaction. Nevertheless, they may be deficient in physical feedback and interactivity that are distinctive of VR experiences. Besides, the types of exergaming can also be categorized into two groups, with or without trainers, based on the presence or absence of a virtual personal trainer concept, which provides feedback and tracks individual progress.

### Subgroup analysis

Subgroup analyses in each sleep domain were conducted based on (i) age, (ii) length of intervention, i.e., intervention period shorter than 8 weeks or not; (iii) type of gaming, i.e., video-based or VR-based; (iv) trainer, i.e., with or without trainer; (v) type of exercise, i.e., aerobic exercise or combined exercise; and (vi) type of participants, i.e., healthy participants or participants with disease.

### Certainty of evidence

The Cochrane Risk of Bias Assessment Tool ROB 2 (2019 version) was used to assess the risk of bias for main outcome measure (sleep quality) (22). The assessment covered the following five domains, including (1) bias due to the randomization process, (2) deviation from intended intervention, (3) missing outcome data, (4) measurement of outcomes, (5) selection of the reported result, and “overall risk of bias” judgment. The bias of each item could be judged as “low risk”, “high risk” or “some concerns”. Two reviewers (KC and YH) assessed the risk of bias independently, conflicts were solved by the third reviewer (ZH).

The Grading of Recommendations Assessment, Development and Evaluation Working Group (GRADE) approach was utilized to assess the certainty of the supporting evidence for each outcome (22).

### Statistical analysis

For studies that provided 95% confidence intervals (CIs), the calculation was performed using the formula outlined in the Cochrane Handbook for Systematic Reviews of Interventions (22).

Studies reported the median and the interquartile range were converted into mean and SD by using the statistical method provided by Luo and Wan (23, 24). For studies with missing SDs, in the absence of essential data for calculation and no response from the authors, the baseline SDs were used as estimates. For RCTs with multiple intervention groups, the data of the experimental groups were combined. For RCTs that used questionnaires where higher scores indicate better sleep quality, the mean scores were multiplied by −1 to ensure that all scales are oriented in the same direction (22).

Standard mean difference (SMD) and 95% confidence interval (95% CI) were used to compare different sleep quality scales. Heterogeneity was assessed using the I² statistic and Cochran's *Q* test, with I² > 50% or *p* < 0.10 indicating substantial heterogeneity. When differences in study characteristics result in heterogeneity (e.g., due to variations in populations, intervention types, or durations), a random-effects model is employed to account for the variability (25). Review Manager 5.4.1(Revman) software was used.

### Role of the funding source

There was no funding source for this review.

## Results

### Characteristics of included studies

**Figure 1:**
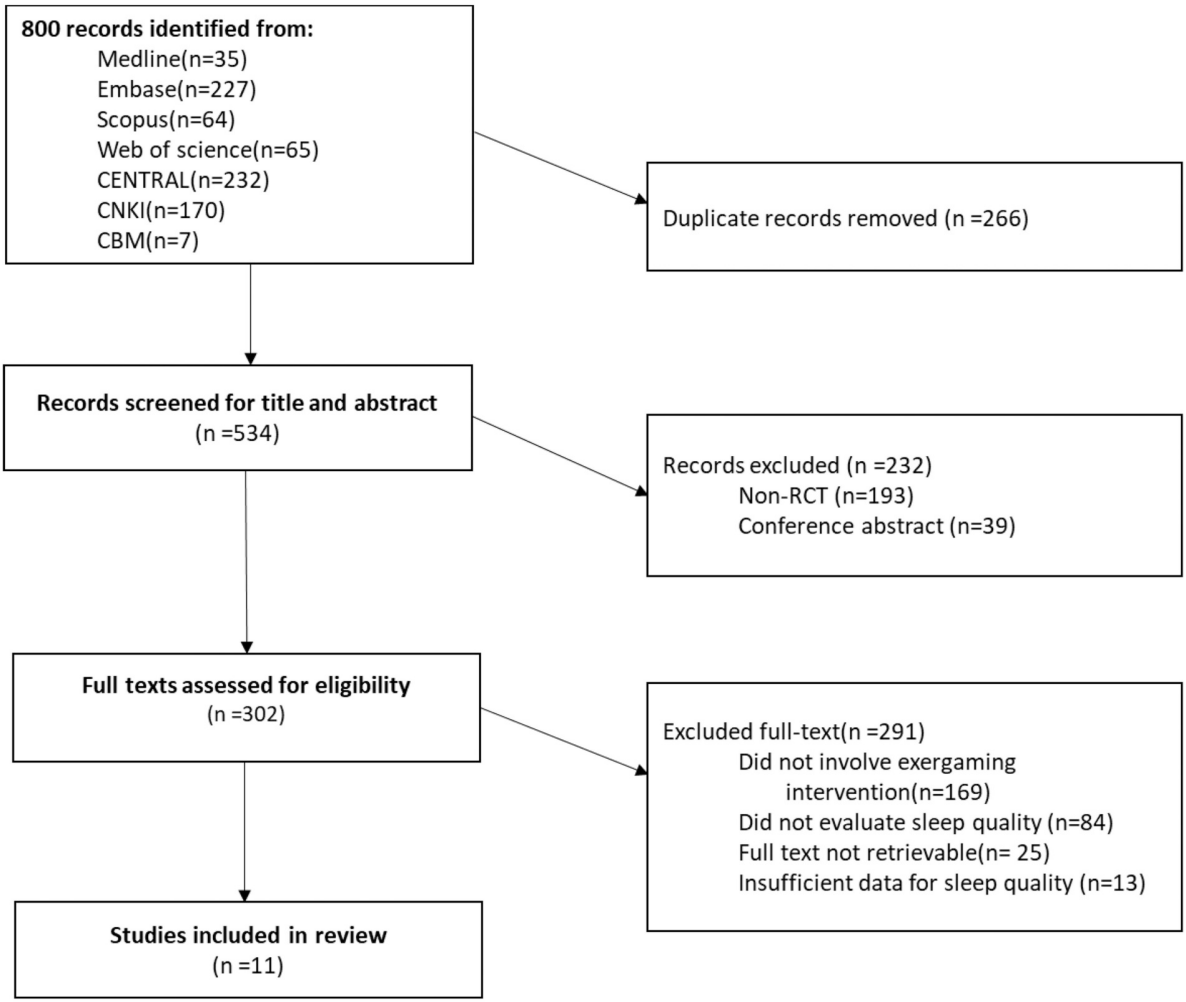
Study selection.

A total of 800 records were identified from the database searches. After removing duplicates, the remaining 534 studies were screened based on their titles and abstracts. Of these, 302 studies focusing on exergaming interventions for improving sleep quality were selected for full-text evaluation. As a result, 11 studies met the eligibility criteria and were included in this systematic review (Figure 1).

The characteristics of the 11 included RCTs are summarized in Table 1. Eight studies solely explored the effects of exergaming vs. no intervention on sleep, while two studies compared exergaming with traditional exercise. Among these included studies, the sample sizes ranged from 19 to 80. Participants represented various age groups, including children, adolescents, adults, and older adults. Most studies (63.6%, 7/11) included approximately equal proportions of male and female participants, except for one study that exclusively involved female participants. Eight studies (72.7%, 8/11) used no exercise as the control group.

**Table 1:** Characteristics of included studies.

Author, year	Country	Setting^a^	Sample size	Mean age	Comorbidity	Male	Intervention intensity		Intervention group				Control group
							Time × frequency	Duration	Gaming type^b^	Exercise type^c^	Trainer^d^	Devices^e^	
Chang, 2014 (26)	China	Care center	9/10	77	N	42%	30 min × 2 sessions/wk	12 wks	Video based	Aerobic	N/A	Stationary devices	No exercise
Farahiyah, 2020 (17)	Malaysia	Public hospital	18/18	23	N	14%	30 min × 3 sessions/wk	6 wks	Video based	Aerobic	No	Stationary devices	No exercise
Lima, 2021 (27)	Brazil	Care center	19/19	68	N	21%	60 min × 3 sessions/wk	6 wks	Video based	Aerobic	No	Stationary devices	No exercise
Tanriverdi, 2021 (16)	Turkey	Public hospital	17/21	13	Y	50%	45 min × 2 sessions/wk	12 wks	Video based	Aerobic	No	Stationary devices	No exercise
Ulas, 2021 (28)^f^	Turkey	School	30/30/20	19	N	45%	30 min × 2 sessions/wk	8 wks	VR based	Aerobic	Yes	VR headset	Traditional exercise; No exercise
Wu, 2022 (29)	China	School	40/40	23	N	58%	30 min × 3 sessions/wk	4 wks	Video based	Combined	Yes	Handheld consoles	No exercise
Masoud, 2023 (30)^g^	Saudi Arabia	Public hospital	22/23	9	Y	56%	60 min × 2 sessions/wk	3 wks	Video based	Aerobic	No	Stationary devices	Advice
Ozdogar, 2023 (31)	Turkey	Public hospital	23/29	41	Y	37%	45 min × 2 sessions/wk	8 wks	Video based	Aerobic	No	Handheld consoles	No exercise
Ahmad 2024 (32)	Egypt	Private clinic	8/8	50	N	15%	30–45 min × 3 sessions/wk	4 wks	VR based	Aerobic	No	VR headset	Traditional exercise
Arias 2024 (33)	Spain	School	44/44	29	N	55%	30 min × 1 session/wk	3 wks	VR based	Aerobic	No	VR headset	Traditional exercise
Lin, 2024 (34)	China	Public hospital	15/14	58	N	0%	60 min × 2 sessions/wk	12 wks	Video based	Combined	Yes	Handheld consoles	No exercise

^a^ Setting: The care center is the elderly care facility that combines medical service and care for the old, which is a continuation and supplement to the hospital. The public hospital is the public institution that provides general medical services.

^b^ VR: virtual reality.

^c^ Combination exercise refers to the combination of aerobic exercise and isometric exercise.

^d^ Exergaming with trainers, also known as Control exergaming, games that capture players’ body movements to control the game. Exergaming without trainers, also known as Workout exergaming, has virtual personal trainers in the game, providing feedback and tracking individual progress.

^e^ Fixed devices: used in a fixed, immovable location, such as home game consoles. Handheld game consoles: portable, movable devices, such as handheld game consoles and touchscreen devices, such as tablets and smartphones, Nintendo Switch.

^f^ In this study, sixty participants were randomly assigned to the VR (*n* = 30) and TE (*n* = 30) groups, and 20 participants formed the control group, not participating in exercise.

^g^ This study through professionals to inform participants of the benefits of physical exercise through lectures and encourages participants to exercise daily.

Geographically, the majority of studies were conducted in Asia (72.2%, 8/11), with one study each from Brazil, Egypt, and Spain. The settings for the studies varied, with 45.5% (5/11) in public hospitals, 18.2% (2/11) in care centers for the elderly and 18.2% (2/11) in schools. One study was conducted in private clinic and another study did not report the study setting. Six studies utilized a two-arm design, three studies had four-arm designs, one study employed a three-arm design, and one study conducted a randomized crossover study. However, only groups using exergaming interventions and their respective control groups were included in the analysis. In these four-arm RCTs, which included two intervention groups and two control groups targeting different populations (e.g., males and females; individuals with and without restless legs syndrome), we combined the groups for the main analysis.

The duration of the exergaming interventions ranged from 3 to 12 weeks, with frequencies of 2–5 sessions per week, and session durations varying between 15 and 60 min. Nine studies focused on aerobic exercises, and two included both aerobic and isometric exercises. For the game types, the exergames in seven studies were exergaming without trainers, in three studies were exergaming with trainers and one study did not report detailed information to identify its exergaming type.

**Figure 2:**
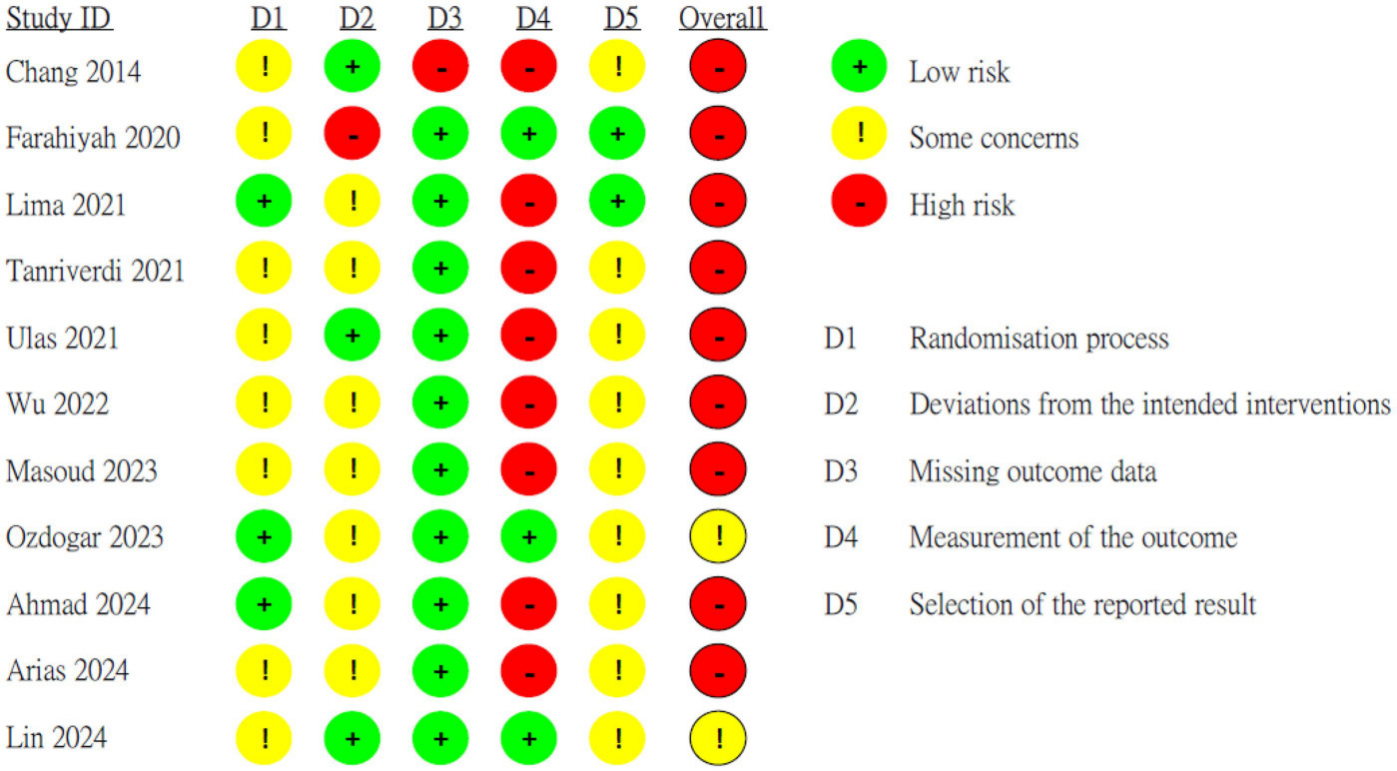
Risk of bias 2 assessment for sleep quality of the included studies.

The Risk of Bias was shown in Figure 2. More details on the GRADE assessment were shown in Table 2. For quality assessment, two studies (18.2%) did not mention their funding sources; however, they did declare no conflict of interest. Seven RCTs (63.6%) reported that they didn't receive the funding. Two studies (18.2%) reported that they received funding support from university research institutions.

**Table 2:** GRADE assessment for each outcome.

Grade assessment			
Sleep quality, exergaming vs. no intervention (No. of Studies: 9)	GRADE criteria	Ratings	Quality of the evidence
	Study design	RCTs	⊕⊕○○ Low
	Risk of bias	Serious (−1)	
	Inconsistency	No, I^2^ = 50%	
	Indirectness	No	
	Imprecision	Serious (−1), *N* = 387 < 400	
	Publication bias	Undetected, <10 studies	
	Other		
Sleep quality, exergaming vs. traditional exercise (No. of studies: 3)	GRADE criteria	Ratings	Quality of the evidence
	Study design	RCTs	⊕⊕○○ Low
	Risk of bias	Serious (−1)	
	Inconsistency	No, I^2^ = 0%	
	Indirectness	No	
	Imprecision	Serious (−1), *N* = 164 < 400	
	Publication bias	Undetected, <10 studies	
	Other		
Sleep latency, exergaming vs. no intervention (No. of studies: 3)	GRADE criteria	Ratings	Quality of the evidence
	Study Design	RCTs	⊕⊕○○ Low
	Risk of Bias	Serious (−1)	
	Inconsistency	No, I^2^ = 0%	
	Indirectness	No	
	Imprecision	Serious (−1), *N* = 119 < 400	
	Publication bias	Undetected, <10 studies	
	Other		
Sleep duration, exergaming vs. no intervention (No. of studies: 3)	GRADE criteria	Ratings	Quality of the Evidence
	Study design	RCTs	⊕⊕○○ Low
	Risk of Bias	Serious (−1)	
	Inconsistency	No, I^2^ = 0%	
	Indirectness	No	
	Imprecision	Serious (−1), *N* = 119 < 400	
	Publication bias	Undetected, <10	
	Other		

### Outcomes assessment scales

Sleep quality was assessed using various quantitative scales, including the Pittsburgh Sleep Quality Index (PSQI), Functional Outcome Sleep Questionnaire (FOSQ), Pediatric Quality of Life Multidimensional Fatigue Scale (Peds QLMFS), and Children's Sleep Habits Questionnaire As shown in Table 3, specific sleep domains scoring systems and cutoffs with each questionnaire for accurate interpretation were provided. It is important to note that, except for the FOSQ and Peds QLMFS, other scales indicate better sleep quality through lower scores.

**Table 3:** Questionnaires summary.

Questionnaire	Assessment type	Sleep domain	No. of items	Total score	Higher/lower score indicated better sleep	Included studies
Pittsburgh sleep quality index (PSQI)	Self-assess and doctor-asses	Time to fall asleep, sleep latency^a^, sleep efficiency^b^, sleep duration^c^, sleep disturbances, use of sleeping medication, daytime dysfunction	19	21	Lower	Chang 2014, Lima 2021, Ulas 2021, Ozdogar 2023, Lin 2024, Ahmad 2024
The Chinese version of PSQI	Self-assess and doctor-assess	Time to fall asleep, sleep duration, sleep efficiency, sleep disturbances	19	21	Lower	Wu 2022
Functional outcome sleep questionnaire	Self-assess	Daytime sleepiness, disorders of excessive sleepiness	30	20	Higher	Farahiyah 2020
Children's sleep habits questionnaire	Parent-assess	Bedtime resistance, sleep onset delay, sleep duration, sleep anxiety, night wakings, parasomnias, sleep-disordered breathing, daytime sleepiness	33	99	Lower	Tanriverdi 2021
Pediatric quality of life multidimensional fatigue scale (PedsQL)	Self-assess	Sleep fatigue	18	100	Higher	Masoud 2023
Karolinska sleep questionnaire	Self-assess	sleep quality, non-restorative sleep, sleep apnea, sleepiness	18	9	Higher	Arias 2024

^a^ Sleep latency is the time it takes to fall asleep after turning the lights ou;.

^b^ Sleep efficiency is the ratio of the total time spent asleep on any given night compared to the total time spent in bed.

^c^ Sleep duration refers to the total amount of sleep obtained, either during the nocturnal sleep episode or across the 24-h period.

### Results of meta-analysis

**Figure 3:**
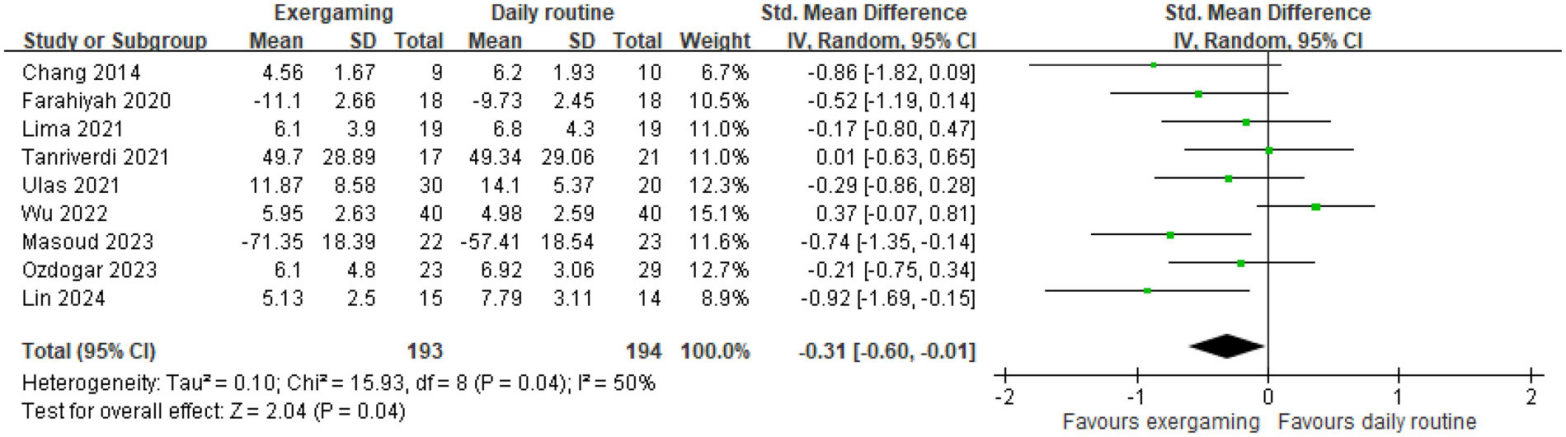
Comparison of exergaming and no intervention on overall sleep quality assessment.

**Figure 4:**
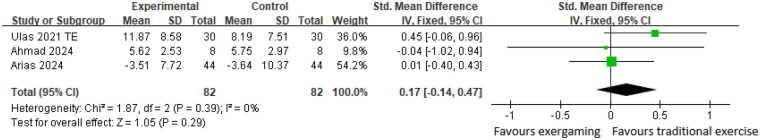
Comparison of exergaming and traditional exercise on overall sleep quality assessment.

Nine RCTs evaluated the effects of exergaming interventions vs. daily routine on overall sleep quality. Seven studies used scales where lower scores indicate better sleep quality, while two (17, 26) used the opposite. Due to substantial heterogeneity (I² = 50%), mixed age range and intervention duration, a random-effects model was applied (Figure 3). Participants in the exergaming groups significantly improve sleep quality compared to controls (SMD = −0.31, 95% CI = −0.60 to −0. 01, k = 9, GRADE = low). Three RCTs comparing exergaming and traditional exercise found no significant difference (SMD = 0.17, 95% CI = −0.14–0.47, k = 3, GRADE = low) (Figure 4). The following are the subgroup analysis of 9 studies comparing exergaming and daily routine.

Nine studies, where lower scores indicate better sleep quality, evaluated the effects of exergaming intervention on sleep across different age groups (Table 4). In participants aged 60 years and older, the intervention led to a significantly greater improvement in sleep compared to controls (SMD = −1.38, 95% CI = −2.75 to −0.00, k = 2). However, in participants aged 18–60 years, no significant effects were observed (SMD = −0.28, 95% CI = −0.63–0.06, k = 7). These findings suggest that the efficacy of exergaming interventions on sleep may vary across age groups, with older adults benefiting the most.

**Table 4:** Effects of exergaming intervention on sleep.

Sleep domains	Overall sleep quality		Sleep latency		Sleep duration	
	k	SMD(95% CI)	k	SMD(95% CI)	k	SMD(95% CI)
Main analysis	**9**	**−0.31** (**−0.60, −0.01)**	3	0.06 (−0.30, 0.42)	3	0.05 (−0.31, 0.41)
Subgroup analysis						
(i) Age						
18–60	7	−0.28 (−0.63, 0.06)	3	0.06 (−0.30, 0.42)	3	0.05 (−0.31, 0.41)
≥ 60	**2**	**−1.38** (**−2.75, −0.00)**				
(ii) Length of intervention						
< 8 weeks	4	−0.24 (−0.77, 0.30)				
≥ 8 weeks	**5**	**−0.35** (**−0.64, −0.06)**	3	0.06 (−0.30, 0.42)	3	0.05 (−0.31, 0.41)
(iii) Type of gaming						
Video based	8	−0.32 (−0.65, 0.02)	3	0.06 (−0.30, 0.42)	3	0.05 (−0.31, 0.41)
VR based	1	−0.29 (−0.86, 0.28)				
(iv) Trainer						
No	**5**	**−0.32** (**−0.60, −0.05)**	2	−0.02 (−0.43, 0.40)	2	0.17 (−0.25, 0.59)
Yes	3	−0.23 (−0.94, 0.49)	1	0.30 (−0.43, 1.03)	1	0.05 (−0.31, 0.41)
N/A	1	−0.86 (−1.82, 0.09)				
(v) Type of exercise						
Aerobic exercise	**7**	**−0.35** (**−0.59, −0.11)**	2	−0.02 (−0.43, 0.40)	2	0.17 (−0.25, 0.59)
Combined	2	−0.24 (−1.50, 1.02)	1	0.30 (−0.43, 1.03)	1	0.05 (−0.31, 0.41)
(vi) Type of participants						
Healthy participants	7^a^	−0.28(−0.66, 0.10)	2^a^	0.10 (−0.51, 0.70)	2^a^	−0.19 (−0.80, 0.41)
With disease	**3** ^a^	**−0.36** (**−0.73, 0.00)**	2^a^	0.06 (−0.39, 0.51)	2^a^	0.17 (−0.28, 0.63)

Bold indicates statistical significance, *P* < 0.05.

^a^ Tanriverdi, 2021 included patients and healthy siblings, who were both randomized into exercise and control groups.

For interventions lasting less than 8 weeks, the effect on sleep was non-significant (SMD = −0.24, 95% CI = −0.77–0.30, k = 4) (Table 4). In contrast, interventions lasting 8 weeks or longer demonstrated statistically significant improvement in sleep (SMD = −0.35, 95% CI = −0.64 to −0.06, k = 5) (Supplementary Figure 1). These findings suggest that longer exergaming interventions may have a more consistent and significant impact on improving sleep.

Participants using video-based exergaming showed a small and non-significant effect on sleep (SMD = −0.32, 95% CI = −0.65–0.02, k = 8) (Table 4). In contrast, the single study using VR-based exergaming demonstrated a larger, but still non-significant effect (SMD = −0.29, 95% CI = −0.86–0.28, k = 1)(Supplementary Figure 1). These results suggest that while both types of gaming show varying effects on sleep, the evidence remains inconclusive, especially for VR-based exergaming. Further research into larger sample sizes and more VR-based interventions is needed to draw conclusions regarding the effectiveness of VR-based exergaming on sleep.

There are two types of exergaming in the nine studies comparing exergaming with daily routines. For exergaming without trainers, participants showed a small but significant improvement in sleep (SMD = −0.32, 95% CI = −0.60 to −0.05, k = 5) (Table 4). In contrast, participants engaging in exergaming with trainers exhibited a small, non-significant improvement (SMD = −0.23, 95% CI = −0.94–0.49, k = 3). Additionally, one study lacked sufficient information to determine the type of exergaming but showed no significant improvement in sleep quality (SMD = −0.86, 95% CI = −1.82–0.09, k = 1) (Supplementary Figure 1).

Participants engaging in aerobic exercise showed a small but significant improvement in sleep (SMD = −0.35, 95% CI = −0.59 to −0.11, k = 7) (Table 4). In contrast, participants involved in combined exercise showed a small, non-significant effect on sleep (SMD = −0.24, 95% CI = −1.5–1.02, k = 2) (Supplementary Figure 1). These findings suggest that aerobic exercise may be more effective in improving sleep than combined exercise types, although further studies with larger sample sizes are needed to confirm these results.

## Discussion

This systematic review evaluated 11 RCTs to assess the effect of exergaming on sleep quality, comparing it with daily routines or traditional exercise. The findings suggest that exergame interventions positively influence sleep quality, particularly for older adults or those with health conditions. Given their accessibility and ease of use, exergames can serve as a self-driven, long-term intervention at home.

The role of exergames should be viewed as supplementary to traditional exercise rather than as a substitute, requiring further investigation to clarify their optimal application. The study conducted by Ulas, which involved 2 intervention groups, indicates that traditional exercise is more effective than exergames in enhancing sleep quality (28). However, exergaming, particularly using virtual reality (VR), increases enjoyment, which could support better adherence to physical activity routine.

Despite these advantages, potential challenges associated with exergaming need attention. A systematic review of the effects of blue light on sleep in young people revealed a reduction in sleep duration and an increase in feelings of tiredness during waking hours (35). Blue light emissions from electronic screens have been demonstrated to disrupt the natural sleep-wake cycle of the brain, thereby affecting sleep quality (36). Furthermore, the increase in screen time and the rising prevalence of game addiction requires close monitoring to ascertain their potential adverse effects on adolescents (37).

Many older adults may not be familiar with new technologies, which can lead to difficulties in using smartphones, tablets, or virtual reality devices. Therefore, it is essential to design age-friendly interfaces that incorporate features such as larger fonts and increased volume to accommodate potential age-related vision and hearing impairments. Additionally, some elderly individuals may find it challenging to engage in high-intensity exergames due to joint issues or muscle degeneration; thus, it is crucial to provide game options tailored to varying levels of physical ability.

Moreover, older adults may encounter obstacles in understanding game rules or remembering operational steps. Consequently, games should offer clear instructions and step-by-step guidance to facilitate a quicker acclimatization. To enhance motivation and adherence among older adults, promoting group activities with family, friends, or community members can significantly improve social interaction and foster a supportive environment for physical engagement.

From our review, exergaming interventions showed significant improvements in sleep quality among older adults aged 60 years and above. Despite exergaming being an innovative approach that provides a safe environment for elderly individuals to engage in physical activities, a systematic review and qualitative meta-synthesis (38) revealed that some older adults are uninterested in exergaming due to age- or health-related factors, such as vision, hearing, motor skills, or cognitive impairments. Moreover, many elderly individuals lack experience with exergaming and worry about their ability to understand and master the required skills (38). However, the self-efficacy fostered by exergaming, along with external support and guidance, can motivate them to participate. A study on the feasibility and practical implications of exergaming for older adults highlighted that participant enjoyed the social interaction of these games and the convenience of performing exergaming at home reduces barriers to keeping participants physically active (39). Yet, the current design of exergames does not target elderly users and often overlooks challenges they may face, such as font size and instructional modes, which can lead to frustration and decrease motivation. Therefore, providing clear, easy-to-understand instructions and helping older adults adapt to exergaming is crucial for its successful introduction to this population.

In this systematic review, we focused solely on sleep-related outcomes reported for the first time following the intervention in the included literature. Among the studies, one recorded data at different time points, while another one conducted follow-up with participants after completing the protocol. In the study on restless legs syndrome, it was noted that at the 8-week follow-up, the effects of exergaming on restless legs syndrome severity, quality of life, sleep quality, and walking capacity were sustained (31). Additionally, in exergaming interventions conducted among children with acute lymphoblastic leukemia, data were recorded at 3 and 5 weeks, consistently demonstrating significant reductions in cancer-related fatigue, along with marked improvements in functional capacity and endurance (26). Furthermore, one study (Ulas) also highlighted changes in participants' lifestyles following the intervention, reporting that this study motivated them to engage in regular exercise.

This review has the following limitations. First, only 11 studies were included in this review, indicating a limited sample size. Of note, there were more than 20 ongoing registered clinical trials related to this topic during our search. Therefore, future updates of this review would be valuable to further explore this topic. Second, sleep quality was not the primary outcome assessed in all included studies. This suggests that the data on sleep quality reported as a secondary outcome are incomplete, as sleep quality may not have been the primary focus of the original study investigation. Third, the use of objective measures was limited. Only one study (16) used polysomnography to evaluate the efficacy of somatosensory games on sleep quality. While self-report data provide valuable insights into individuals’ perceptions of their sleep, they inherently lack the ability to capture physiological effects and variations in sleep architecture. This limitation can lead to an incomplete understanding of the true impact of interventions on sleep quality. Therefore, future studies should prioritize the combination of subjective and objective measures. By integrating both types of assessments, researchers can achieve a more comprehensive evaluation of sleep outcomes, ultimately leading to better-informed conclusions and recommendations regarding interventions aimed at improving sleep quality.

Among the 11 included studies, 9 of them exhibited a high risk of bias, which contributed to an overall low certainty of evidence according to the GRADE assessment. The main sources of bias identified in these studies included the lack of blinding and unclear randomization procedures. These shortcomings underscore the necessity for more rigorous methodologies in future research. To enhance the quality and reliability of RCTs assessing the effects of exergaming interventions on sleep quality, future studies should implement blinding for both participants and outcome assessors to minimize potential bias, transparent and detailed descriptions of randomization processes should be provided to ensure the integrity of the random assignment of participants. Besides, researchers should consider larger and more diverse samples to enhance the generalizability of the findings and reduce heterogeneity among results. Clear reporting of funding sources and potential conflicts of interest should be maintained to bolster the credibility of the research.

The findings of this systematic review on exergaming and sleep quality indicate that exergames may represent a feasible option for maintaining physical activity and positively influencing sleep health. The widespread use of video games raises important implications for public health, especially in the context of the COVID-19 pandemic and the emergence of AI. It is crucial to develop novel and diverse intervention guidelines for specific sleep disorders, thereby maximizing the benefits of exergaming for different sleep disorders in the future.

## Future clinical and research suggestions

Considering that exergaming offers a feasible and engaging method for improving sleep quality, particularly through home-based physical activities, clinicians and healthcare providers should familiarize themselves with this intervention and encourage individuals, especially older adults, to incorporate exergaming into their routines. Given that exergaming is an accessible tool for enhancing sleep quality, it can serve as an effective and enjoyable alternative to traditional exercise methods. Future studies should focus on assessing the long-term benefits of exergaming interventions and exploring their potential in various populations, particularly older adults, to ensure their broader application in healthcare. In addition, exergaming interventions should be tailored to the specific needs of elderly users, addressing issues such as font size, clarity of instructions, and ease of use to maximize participation and adherence. As the design of exergames continues to evolve, it is crucial that these tools are made user-friendly and adaptable to different age groups and physical abilities.

## Conclusion

Exergame interventions positively influence sleep quality, particularly for older adults or those with health conditions. Given their accessibility and ease of use, exergames can serve as a self-driven, long-term intervention at home. Future research should focus on optimizing these interventions to maximize their effectiveness and promote widespread adoption.

## Data Availability

The original contributions presented in the study are included in the article/Supplementary Material, further inquiries can be directed to the corresponding author.
