# Supplementary material for: The effects of exergaming on sleep quality: a systematic review and meta-analysis of randomized controlled trials
Source: Front Digit Health. 2026 Feb 9;8:1699626. doi: 10.3389/fdgth.2026.1699626 (PMC12926418; doi:10.3389/fdgth.2026.1699626)

Supplementary Table 1. Full Search Terms for Databases

<u>**Databases**</u>
**Medline**
#1 exp gamification/ or exp game/<br>#2 Exergame.mp.<br>#3 exp virtual reality/ or virtual reality.mp.<br>#4 nintendo wii.mp. or exp video game/<br>#5 xbox Kinect.mp.<br>#6 wii fit.mp.<br>#7 Kinect.mp.<br>#8 Exergamings.mp. or exp exergaming/<br>#9 #1 or #2 or #3 or #4 or #5 or #6 or #7 #or 8<br>#10 exp sleep latency/ or exp circadian rhythm sleep disorder/ or exp sleep time/ or exp sleep quality/ or exp central sleep apnea syndrome/ or exp shift work sleep disorder/ or exp sleep study/ or exp advanced sleep phase syndrome/ or exp sleep efficiency/ or exp Leeds Sleep Evaluation Questionnaire/ or exp sleep disorder assessment/ or exp sleep depth/ or exp sleep disorder/ or exp delayed sleep phase syndrome/ or exp night sleep/ or exp pediatric sleep questionnaire/ or exp Pittsburgh Sleep Quality Index/ or exp medical outcomes study sleep scale/ or exp sleep waking cycle/ or sleep/ or exp sleep questionnaire/<br>#11 Randomized controlled trials.mp. or exp "randomized controlled trial (topic)"/<br>#12 Controlled clinical trial.mp. or exp controlled clinical trial/<br>#13 Clinical trial.mp. or exp clinical trial/<br>#14 #11 or #12 or #13<br>#15 #9 and #10 and #14
**Embase**
#1 exp gamification/ or exp game/<br>#2 Exergame.mp.<br>#3 exp virtual reality/ or virtual reality.mp.<br>#4 nintendo wii.mp. or exp video game/<br>#5 xbox Kinect.mp.<br>#6 wii fit.mp.<br>#7 Kinect.mp.<br>#8 Exergamings.mp. or exp exergaming/<br>#9 #1 or #2 or #3 or #4 or #5 or #6 or #7 #or 8<br>#10 exp sleep latency/ or exp circadian rhythm sleep disorder/ or exp sleep time/ or exp sleep quality/ or exp central sleep apnea syndrome/ or exp shift work sleep disorder/ or exp sleep study/ or exp advanced sleep phase syndrome/ or exp sleep efficiency/ or exp Leeds Sleep Evaluation Questionnaire/ or exp sleep disorder assessment/ or exp sleep depth/ or exp sleep disorder/ or exp delayed sleep phase syndrome/ or exp night sleep/ or exp pediatric sleep questionnaire/ or exp Pittsburgh Sleep Quality Index/ or exp medical outcomes study sleep scale/ or exp sleep waking cycle/ or sleep/ or exp sleep questionnaire/<br>#11 Randomized controlled trials.mp. or exp "randomized controlled trial (topic)"/<br>#12 Controlled clinical trial.mp. or exp controlled clinical trial/<br>#13 Clinical trial.mp. or exp clinical trial/<br>#14 #11 or #12 or #13<br>#15 #9 and #10 and #14
**Scopus**
#1 (exergam OR virtual AND reality OR nintendo AND wii OR xbox AND kinect OR wii AND fit OR kinect OR wii) AND (sleep AND quality OR sleep AND duration OR pittsburgh AND sleep AND quality AND index OR polysomnography OR actigraphy) AND (randomized AND controlled AND trials OR controlled AND clinical AND trial OR clinical AND trial OR placebo OR blind* OR random* OR trial)
**Web of Science**
#1 ((((((ALL=(exergam)) OR ALL=(virtual reality)) OR ALL=(nintendo wii)) OR ALL=(xbox Kinect)) OR ALL=(wii fit)) OR ALL=(Kinect Wii)) OR ALL=(Exergamings)<br>#2 ((((ALL=(Sleep quality)) OR ALL=(Pittsburgh sleep quality index)) OR ALL=(polysomnography)) OR ALL=(Actigraphy)) OR ALL=(Sleep Duration)<br>#3 ((((ALL=(Randomized controlled trials)) OR ALL=(Controlled clinical trial)) OR ALL=(Clinical trial)) OR ALL=(placebo)) OR ALL=(Random*)<br>#4 #1 AND #2 AND #3
**Central Cochrane**
#1 (Exergam) (Word variations have been searched)<br>#2 (virtual reality) OR (nintendo wii) OR (xbox Kinect) OR (wii fit) OR (Kinect) (Word variations have been searched)<br>#3 MeSH descriptor: [Exergaming] explode all trees<br>#4 #1 OR #2 OR #3<br>#5 (sleep) OR (insomnia) OR (polysomnography) OR (Actigraphy) OR (Pittsburgh sleep quality index) (Word variations have been searched)<br>#6 MeSH descriptor: [Sleep Quality] explode all trees<br>#7 #5 OR #6<br>#8 (Randomized controlled trials) OR (Controlled clinical trial) OR (Clinical trial) OR (blind) AND (placebo) (Word variations have been searched)<br>#9 #4 AND #7 AND #8
**CNKI**
#1 ((运动游戏 OR 虚拟现实 OR 任天堂 OR xbox Kinect)) and 全部:(睡眠质量 OR 睡眠时长 OR 匹兹堡睡眠质量指数 OR 多导睡眠图 OR 活动记录仪)) and 全部:((随机对照试验 OR 对照临床试验 OR 临床试验 OR 安慰剂))
**CBM**

#1 "Exergam"[全部字段:智能] OR "virtual"[常用字段:智能] AND "reality"[常用字段:智能] OR "nintendo"[常用字段:智能] AND "wii"[常用字段:智能] OR "xbox"[常用字段:智能] AND "Kinect"[常用字段:智能] OR "wii"[常用字段:智能] AND "fit"[常用字段:智能] OR "Kinect"[常用字段:智能] AND "Wii"[常用字段:智能]
#2 "sleep"[常用字段:智能] AND "quality"[常用字段:智能] OR "Pittsburgh"[常用字段:智能] AND "sleep"[常用字段:智能] AND "quality"[常用字段:智能] AND "index"[常用字段:智能] OR "polysomnography"[常用字段:智能] OR "Actigraphy"[常用字段:智能] OR "Sleep"[常用字段:智能] AND "Duration"[常用字段:智能]
#3 "Randomized"[常用字段:智能] AND "controlled"[常用字段:智能] AND "trials"[常用字段:智能] OR "Controlled"[常用字段:智能] AND "clinical"[常用字段:智能] AND "trial"[常用字段:智能] OR "Clinical"[常用字段:智能] AND "trial"[常用字段:智能]
#4 #1 AND #2 AND #3

Supplementary Figure 1. Subgroup Analysis for Sleep Quality

a. Age

Age 18-60

Study or Subgroup	Experimental Mean	Experimental SD	Experimental Total	Control Mean	Control SD	Control Total	Weight	Std. Mean Difference IV, Random, 95% CI
Farahiyah 2020	-11.1	2.66	18	-9.73	2.45	18	13.0%	-0.52 [-1.19, 0.14]
Tanriverdi 2021	49.7	28.89	17	49.34	29.06	21	13.5%	0.01 [-0.63, 0.65]
Ulas 2021	11.87	8.58	30	14.1	5.37	20	15.0%	-0.29 [-0.86, 0.28]
Wu 2022	5.95	2.63	40	4.98	2.59	40	17.8%	0.37 [-0.07, 0.81]
Masoud 2023	-71.35	18.39	22	-57.41	18.54	23	14.2%	-0.74 [-1.35, -0.14]
Ozdogar 2023	6.1	4.8	23	6.92	3.06	29	15.4%	-0.21 [-0.75, 0.34]
Lin 2024	5.13	2.5	15	7.79	3.11	14	11.1%	-0.92 [-1.69, -0.15]
**Total (95% CI)**			**165**			**165**	**100.0%**	**-0.28 [-0.63, 0.06]**

Heterogeneity: Tau² = 0.12; Chi² = 14.17, df = 6 (P = 0.03); I² = 58%
Test for overall effect: Z = 1.61 (P = 0.11)

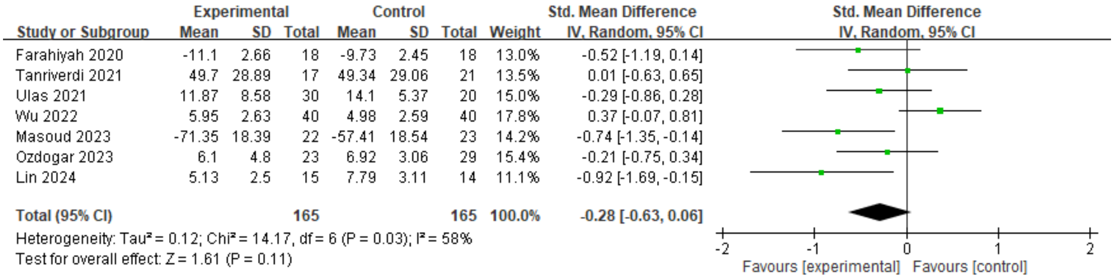

Age ⩾60

Study or Subgroup	Experimental Mean	Experimental SD	Experimental Total	Control Mean	Control SD	Control Total	Weight	Mean Difference IV, Fixed, 95% CI
Chang 2014	4.56	1.67	9	6.2	1.93	10	72.2%	-1.64 [-3.26, -0.02]
Lima 2021	6.1	3.9	19	6.8	4.3	19	27.8%	-0.70 [-3.31, 1.91]
**Total (95% CI)**			**28**			**29**	**100.0%**	**-1.38 [-2.75, -0.00]**

Heterogeneity: Chi² = 0.36, df = 1 (P = 0.55); I² = 0%
Test for overall effect: Z = 1.96 (P = 0.05)

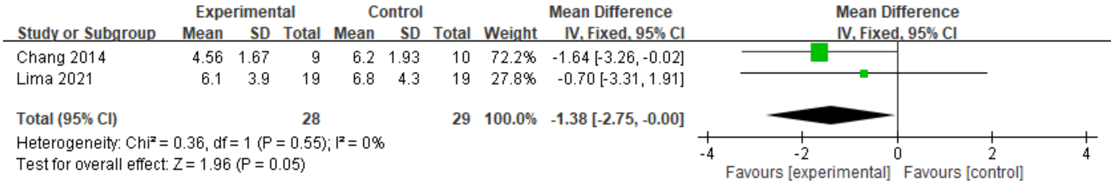

b. Length of Intervention

< 8 weeks

Study or Subgroup	Experimental Mean	Experimental SD	Experimental Total	Control Mean	Control SD	Control Total	Weight	Std. Mean Difference IV, Random, 95% CI
Farahiyah 2020	-11.1	2.66	18	-9.73	2.45	18	23.0%	-0.52 [-1.19, 0.14]
Lima 2021	6.1	3.9	19	6.8	4.3	19	23.7%	-0.17 [-0.80, 0.47]
Wu 2022	5.95	2.63	40	4.98	2.59	40	28.8%	0.37 [-0.07, 0.81]
Masoud 2023	-71.35	18.39	22	-57.41	18.54	23	24.5%	-0.74 [-1.35, -0.14]
**Total (95% CI)**			**99**			**100**	**100.0%**	**-0.24 [-0.77, 0.30]**

Heterogeneity: Tau² = 0.21; Chi² = 10.14, df = 3 (P = 0.02); I² = 70%
Test for overall effect: Z = 0.87 (P = 0.39)

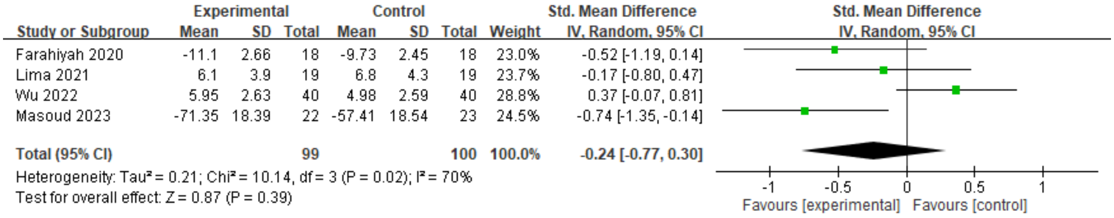

⩾ 8 weeks

Study or Subgroup	Experimental Mean	Experimental SD	Experimental Total	Control Mean	Control SD	Control Total	Weight	Std. Mean Difference IV, Fixed, 95% CI
Chang 2014	4.56	1.67	9	6.2	1.93	10	9.5%	-0.86 [-1.82, 0.09]
Tanriverdi 2021	49.7	28.89	17	49.34	29.06	21	21.0%	0.01 [-0.63, 0.65]
Ulas 2021	11.87	8.58	30	14.1	5.37	20	26.5%	-0.29 [-0.86, 0.28]
Ozdogar 2023	6.1	4.8	23	6.92	3.06	29	28.5%	-0.21 [-0.75, 0.34]
Lin 2024	5.13	2.5	15	7.79	3.11	14	14.4%	-0.92 [-1.69, -0.15]
**Total (95% CI)**			**94**			**94**	**100.0%**	**-0.35 [-0.64, -0.06]**

Heterogeneity: Chi² = 4.75, df = 4 (P = 0.31); I² = 16%
Test for overall effect: Z = 2.33 (P = 0.02)

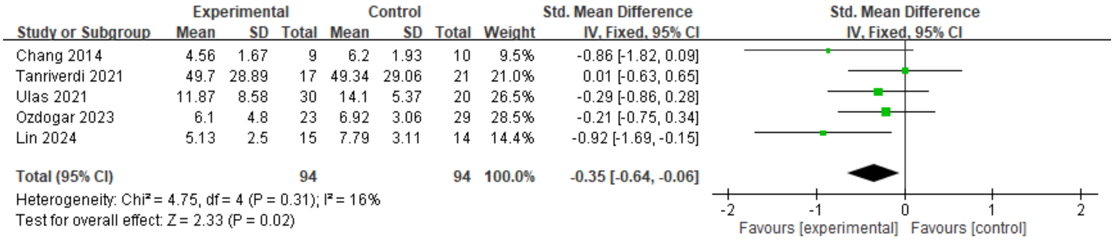

c. Type of Gaming

Video-based

Study or Subgroup	Experimental Mean	Experimental SD	Experimental Total	Control Mean	Control SD	Control Total	Weight	Std. Mean Difference IV, Random, 95% CI
Chang 2014	4.56	1.67	9	6.2	1.93	10	8.1%	-0.86 [-1.82, 0.09]
Farahiyah 2020	-11.1	2.66	18	-9.73	2.45	18	12.1%	-0.52 [-1.19, 0.14]
Lima 2021	6.1	3.9	19	6.8	4.3	19	12.6%	-0.17 [-0.80, 0.47]
Tanriverdi 2021	49.7	28.89	17	49.34	29.06	21	12.6%	0.01 [-0.63, 0.65]
Wu 2022	5.95	2.63	40	4.98	2.59	40	16.5%	0.37 [-0.07, 0.81]
Masoud 2023	-71.35	18.39	22	-57.41	18.54	23	13.2%	-0.74 [-1.35, -0.14]
Ozdogar 2023	6.1	4.8	23	6.92	3.06	29	14.3%	-0.21 [-0.75, 0.34]
Lin 2024	5.13	2.5	15	7.79	3.11	14	10.4%	-0.92 [-1.69, -0.15]
**Total (95% CI)**			**163**			**174**	**100.0%**	**-0.32 [-0.65, 0.02]**

Heterogeneity: Tau² = 0.13; Chi² = 15.89, df = 7 (P = 0.03); I² = 56%
Test for overall effect: Z = 1.84 (P = 0.07)

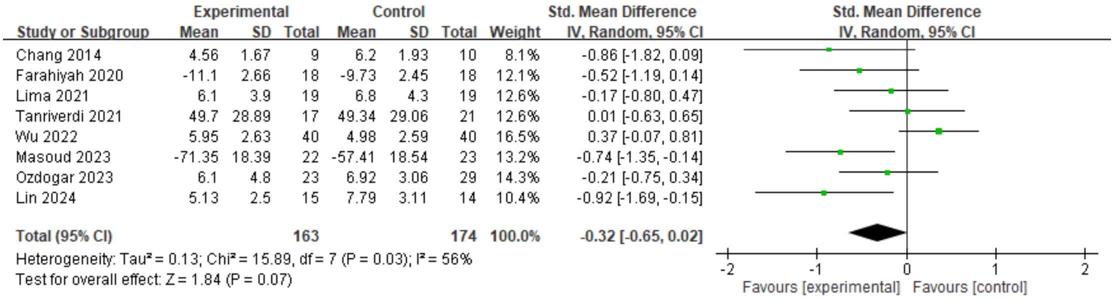

VR-based: only 1 study Ulas

d. Trainer

Without trainer

Study or Subgroup	Experimental Mean	Experimental SD	Experimental Total	Control Mean	Control SD	Control Total	Weight	Std. Mean Difference IV, Fixed, 95% CI
Farahiyah 2020	-11.1	2.66	18	-9.73	2.45	18	17.1%	-0.52 [-1.19, 0.14]
Lima 2021	6.1	3.9	19	6.8	4.3	19	18.7%	-0.17 [-0.80, 0.47]
Tanriverdi 2021	49.7	28.89	17	49.34	29.06	21	18.5%	0.01 [-0.63, 0.65]
Masoud 2023	-71.35	18.39	22	-57.41	18.54	23	20.6%	-0.74 [-1.35, -0.14]
Ozdogar 2023	6.1	4.8	23	6.92	3.06	29	25.1%	-0.21 [-0.75, 0.34]
**Total (95% CI)**			**99**			**110**	**100.0%**	**-0.32 [-0.60, -0.05]**

Heterogeneity: Chi² = 3.64, df = 4 (P = 0.46); I² = 0%
Test for overall effect: Z = 2.30 (P = 0.02)

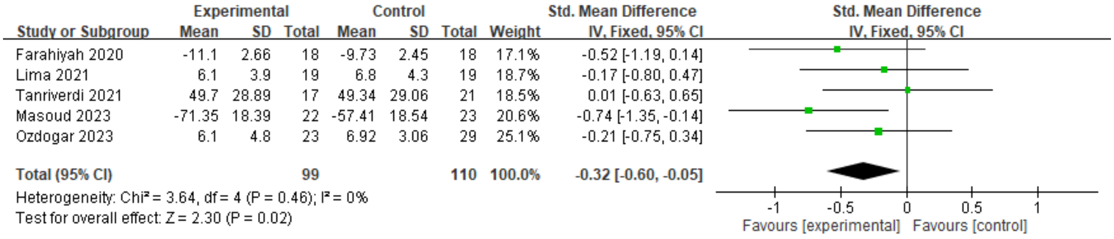

With trainer

Study or Subgroup	Experimental Mean	Experimental SD	Experimental Total	Control Mean	Control SD	Control Total	Weight	Std. Mean Difference IV, Random, 95% CI
Ulas 2021	11.87	8.58	30	14.1	5.37	20	34.0%	-0.29 [-0.86, 0.28]
Wu 2022	5.95	2.63	40	4.98	2.59	40	37.2%	0.37 [-0.07, 0.81]
Lin 2024	5.13	2.5	15	7.79	3.11	14	28.8%	-0.92 [-1.69, -0.15]
**Total (95% CI)**			**85**			**74**	**100.0%**	**-0.23 [-0.94, 0.49]**

Heterogeneity: Tau² = 0.31; Chi² = 9.02, df = 2 (P = 0.01); I² = 78%
Test for overall effect: Z = 0.63 (P = 0.53)

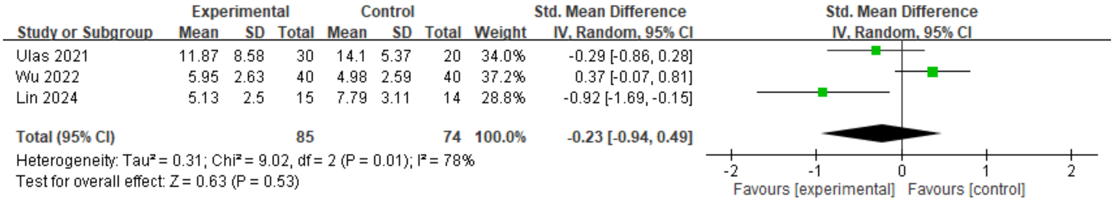

e. Type of Exercise

Aerobic exercise

Study or Subgroup	Experimental Mean	Experimental SD	Experimental Total	Control Mean	Control SD	Control Total	Weight	Std. Mean Difference IV, Fixed, 95% CI
Chang 2014	4.56	1.67	9	6.2	1.93	10	6.3%	-0.86 [-1.82, 0.09]
Farahiyah 2020	-11.1	2.66	18	-9.73	2.45	18	13.0%	-0.52 [-1.19, 0.14]
Lima 2021	6.1	3.9	19	6.8	4.3	19	14.2%	-0.17 [-0.80, 0.47]
Tanriverdi 2021	49.7	28.89	17	49.34	29.06	21	14.1%	0.01 [-0.63, 0.65]
Ulas 2021	11.87	8.58	30	14.1	5.37	20	17.8%	-0.29 [-0.86, 0.28]
Ozdogar 2023	6.1	4.8	23	6.92	3.06	29	19.1%	-0.21 [-0.75, 0.34]
Masoud 2023	-71.35	18.39	22	-57.41	18.54	23	15.6%	-0.74 [-1.35, -0.14]
**Total (95% CI)**			**138**			**140**	**100.0%**	**-0.35 [-0.59, -0.11]**

Heterogeneity: Chi² = 4.84, df = 6 (P = 0.56); I² = 0%
Test for overall effect: Z = 2.88 (P = 0.004)

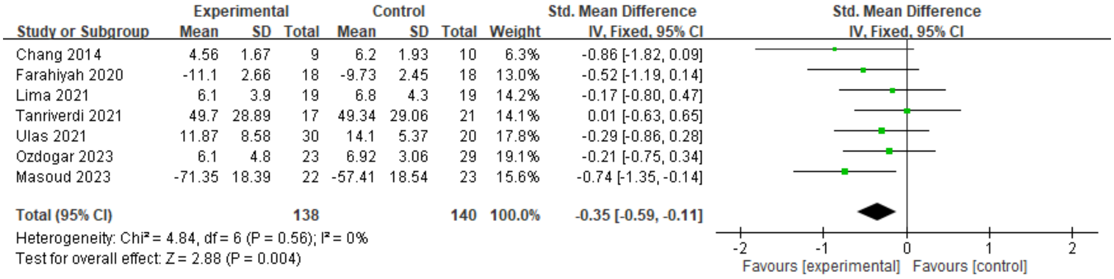

Combined exercise

	Experimental			Control				Std. Mean Difference
Study or Subgroup	Mean	SD	Total	Mean	SD	Total	Weight	IV, Random, 95% CI
Wu 2022	5.95	2.63	40	4.98	2.59	40	53.1%	0.37 [-0.07, 0.81]
Lin 2024	5.13	2.5	15	7.79	3.11	14	46.9%	-0.92 [-1.69, -0.15]
**Total (95% CI)**			**55**			**54**	**100.0%**	**-0.24 [-1.50, 1.02]**

Heterogeneity: Tau² = 0.73; Chi² = 8.06, df = 1 (P = 0.005); I² = 88%
Test for overall effect: Z = 0.37 (P = 0.71)

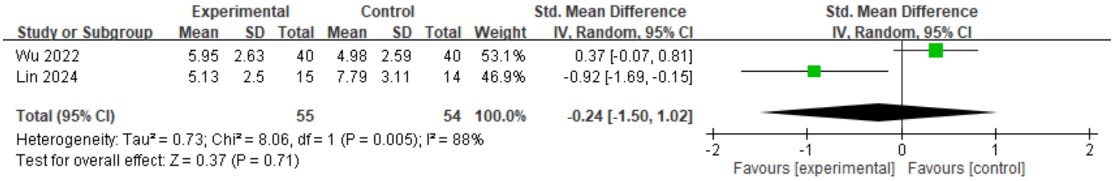

f. Type of Participants

Healthy participants

	Experimental			Control				Std. Mean Difference
Study or Subgroup	Mean	SD	Total	Mean	SD	Total	Weight	IV, Random, 95% CI
Chang 2014	4.56	1.67	9	6.2	1.93	10	10.1%	-0.86 [-1.82, 0.09]
Farahiyah 2020	-11.1	2.66	18	-9.73	2.45	18	15.0%	-0.52 [-1.19, 0.14]
Lima 2021	6.1	3.9	19	6.8	4.3	19	15.6%	-0.17 [-0.80, 0.47]
Tanriverdi 2021	52.33	29.28	6	49.52	29.28	8	8.8%	0.09 [-0.97, 1.15]
Ulas 2021	11.87	8.58	30	14.1	5.37	20	17.2%	-0.29 [-0.86, 0.28]
Wu 2022	5.95	2.63	40	4.98	2.59	40	20.3%	0.37 [-0.07, 0.81]
Lin 2024	5.13	2.5	15	7.79	3.11	14	13.0%	-0.92 [-1.69, -0.15]
**Total (95% CI)**			**137**			**129**	**100.0%**	**-0.28 [-0.66, 0.10]**

Heterogeneity: Tau² = 0.13; Chi² = 12.85, df = 6 (P = 0.05); I² = 53%
Test for overall effect: Z = 1.45 (P = 0.15)

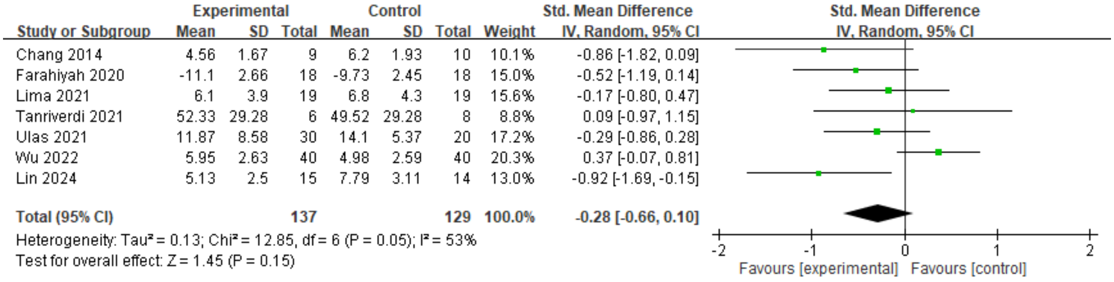

Participants with disease

	Experimental			Control				Std. Mean Difference
Study or Subgroup	Mean	SD	Total	Mean	SD	Total	Weight	IV, Fixed, 95% CI
Tanriverdi 2021	48.27	30.12	11	49.23	30.12	13	20.4%	-0.03 [-0.83, 0.77]
Ozdogar 2023	6.1	4.8	23	6.92	3.06	29	43.7%	-0.21 [-0.75, 0.34]
Masoud 2023	-71.35	18.39	22	-57.41	18.54	23	35.8%	-0.74 [-1.35, -0.14]
**Total (95% CI)**			**56**			**65**	**100.0%**	**-0.36 [-0.73, 0.00]**

Heterogeneity: Chi² = 2.47, df = 2 (P = 0.29); I² = 19%
Test for overall effect: Z = 1.96 (P = 0.05)

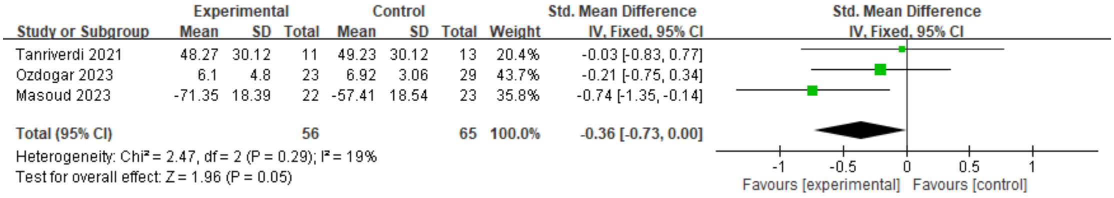

Supplement: Supplementary file 1 [file Datasheet1.pdf]
